# Local p‐ and n‐Type Doping of an Oxide Semiconductor via Electric‐Field‐Driven Defect Migration

**DOI:** 10.1002/advs.202506629

**Published:** 2025-09-18

**Authors:** Jiali He, Ursula Ludacka, Kasper A. Hunnestad, Didrik R. Småbråten, Konstantin Shapovalov, Per Erik Vullum, Constantinos Hatzoglou, Donald M. Evans, Erik D. Roede, Zewu Yan, Edith Bourret, Sverre M. Selbach, David Gao, Jaakko Akola, Dennis Meier

**Affiliations:** ^1^ Department of Materials Science and Engineering NTNU Norwegian University of Science and Technology Trondheim 7034 Norway; ^2^ Department of Electronic Systems NTNU Norwegian University of Science and Technology Trondheim 7034 Norway; ^3^ Department of Sustainable Energy Technology SINTEF Industry Oslo 0373 Norway; ^4^ Theoretical Materials Physics, Q‐MAT University of Liège Liège B‐4000 Belgium; ^5^ SINTEF Industry Trondheim 7034 Norway; ^6^ Department of Physics NTNU Norwegian University of Science and Technology Trondheim 7491 Norway; ^7^ Department of Physics ETH Zürich Zürich 8093 Switzerland; ^8^ Materials Sciences Division Lawrence Berkeley National Laboratory Berkeley CA 94720 USA; ^9^ Nanolayers Research Computing London NW9 6PL UK; ^10^ Computational Physics Laboratory Tampere University Tampere FI‐33014 Finland; ^11^ Faculty of Physics University of Duisburg‐Essen 47057 Duisburg Germany

**Keywords:** Anti‐Frenkel defects, Ferroelectric oxides, Functionalization of oxide semiconductors, Oxide electronics, Oxygen defects

## Abstract

Layered oxides exhibit high ionic mobility and chemical flexibility, attracting interest as cathode materials for lithium‐ion batteries and the pairing of hydrogen production and carbon capture. Recently, layered oxides emerged as highly tunable semiconductors. For example, by introducing anti‐Frenkel defects, the electronic hopping conductance in hexagonal manganites is increased locally by orders of magnitude. Here, local acceptor and donor doping in Er(Mn,Ti)O_3_ is demonstrated, facilitated by the controlled splitting of anti‐Frenkel defects under applied d.c. voltage. By combining density functional theory calculations, scanning probe microscopy, atom probe tomography, and scanning transmission electron microscopy, it is shown that the oxygen defects can readily be moved through the layered crystal structure, leading to nano‐sized interstitial‐rich (p‐type) and vacancy‐rich (n‐type) regions. The resulting pattern is comparable to dipolar npn‐junctions and stable on the timescale of days. These findings reveal the possibility of temporarily functionalizing oxide semiconductors at the nanoscale, giving additional opportunities for the field of oxide electronics and the development of transient electronics in general.

## Graphical abstract

Local acceptor and donor doping is achieved in a semiconducting oxide by controlled splitting of oxygen interstitial‐vacancy pairs under applied d.c. voltage. The separated oxygen defects are demonstrated to form interstitial‐rich (p‐type) and vacancy‐rich (n‐type) regions, comparable to dipolar npn‐junctions, enabling transient functionalization.

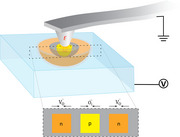

## 1 Introduction

Precise control of defects in solids is essential to introduce and tune functional properties. A classical example is p‐ and n‐type semiconductors, which owe their electronic properties to the introduction of acceptor and donor atoms, respectively, representing the backbone of modern information and communication technology.^[^ ^1^, ^2^ ^]^ Oxide semiconductors are a special sub‐category, that offer an outstanding tunability when it comes to defects.^[^ ^3^ ^]^ In these materials, oxygen defects (i.e., vacancies and interstitials) can readily be used to manipulate the mechanical,^[^ ^4^ ^]^ electric,^[^ ^5^ ^]^ and magnetic properties,^[^ ^6^ ^]^ giving rise to additional degrees of freedom not available in conventional semiconductors. The flexibility of oxides has motivated the design of innovative device architectures for next‐generation nanoelectronics and spintronics. Opportunities range from oxide‐based memristors for neuromorphic computing^[^ ^7^, ^8^, ^9^ ^]^ to energy‐efficient spin filters and logical gates.^[^ ^10^, ^11^ ^]^

In recent years, tremendous progress has been made in controlling oxygen defects in oxide semiconductors.^[^ ^12^, ^13^, ^14^, ^15^ ^]^ An intriguing example is SrTiO_3_ superlattices with oxygen vacancy gradients, which were achieved by tuning the oxygen pressure during synthesis, demonstrating atomic‐scale control.^[^ ^16^ ^]^ Importantly, oxygen defects are particularly versatile and can be generated and manipulated even after a material has been synthesized. For instance, it has been demonstrated that by annealing LuFe_2_O_4_ in an oxygen‐rich atmosphere, oxygen interstitials can be created at will, altering both the material's charge ordering and spin configuration.^[^ ^17^ ^]^ Furthermore, local manipulation of oxygen defects is possible using a broad range of stimuli, including intense light fields, X‐rays, focused electron beams, and electrical voltage.^[^ ^12^, ^18^ ^]^ Examples are the formation of a 2D electron gas at the surface of KTaO_3_ under irradiation of intense UV light^[^ ^19^ ^]^ and the electric‐field‐driven metal‐insulator transition in VO_2_, which relates to the creation and annihilation of oxygen vacancies.^[^ ^20^ ^]^ Depending on the material and type of defects, the induced changes can persist for years, which is of interest in the development of, e.g., circuitry and memory technology,^[^ ^21^ ^]^ or disappear over time, as required for transient electronics.^[^ ^22^ ^]^ For a broader overview of this rapidly evolving field, the interested reader is referred to recent review articles, e.g., ref. [23] and ref. [24].

In addition to just individual oxygen vacancies or interstitials, more complex defect arrangements can be applied to control the material's physical properties. Schottky defects in MgO, consisting of paired magnesium and oxygen vacancies, were found to exhibit better stability and a lower migration energy than isolated vacancies.^[^ ^25^ ^]^ In cubic Lu_2_O_3_, the formation of oxygen vacancy‐vacancy pairs introduces additional energy levels within the band gap, contributing to the coloration and optical absorption properties.^[^ ^26^ ^]^ Recently, in 0.2% Ti‐doped erbium manganite, Er(Mn,Ti)O_3_, anion interstitial‐vacancy pairs (anti‐Frenkel defects) were shown to locally enhance the conductance by up to four orders of magnitude, enabling conductivity control at the nanoscale.^[^ ^27^ ^]^

Motivated by this pronounced correlation between oxygen defects and electronic transport properties in an oxide semiconductor, and its remarkable flexibility when it comes to accommodating oxygen defects in general, we focus on layered hexagonal Er(Mn,Ti)O_3_ as the model system in this work. With our spatio‐temporal experiments, we go beyond just injecting interstitial‐vacancy pairs, exploring how different combinations of voltage and exposure time can be utilized to split such pairs and control the migration of positively and negatively charged oxygen defects. We demonstrate that nano‐sized interstitial‐rich (p‐type) and vacancy‐rich (n‐type) regions can be created on demand and develop a microscopic model for the defect formation process.

Er(Mn,Ti)O_3_ is a ferroelectric p‐type semiconductor,^[^ ^28^ ^]^ which has been intensively studied with respect to its multiferroic properties, as well as the emergence of unusual ferroelectric domains and functional domain walls.^[^ ^29^, ^30^, ^31^, ^32^, ^33^, ^34^ ^]^ Most importantly for this work, the system exhibits an outstanding chemical flexibility that can be leveraged for property engineering via oxygen defects.^[^ ^28^, ^35^, ^36^, ^37^, ^38^ ^]^ In contrast to perovskite‐type oxides, oxygen vacancies and interstitials play an equally significant role in the electronic transport properties of hexagonal manganites.^[^ ^39^ ^]^ At the bulk level, it is established that both n‐ and p‐type behavior can be induced on demand,^[^ ^40^ ^]^ facilitated by the layered and rather open hexagonal crystal structure, where atoms are less densely packed than in perovskite systems.

## 2 Spatio‐Temporal Evolution of Electric‐Field‐Induced Oxygen Defects

**Figure 1:**
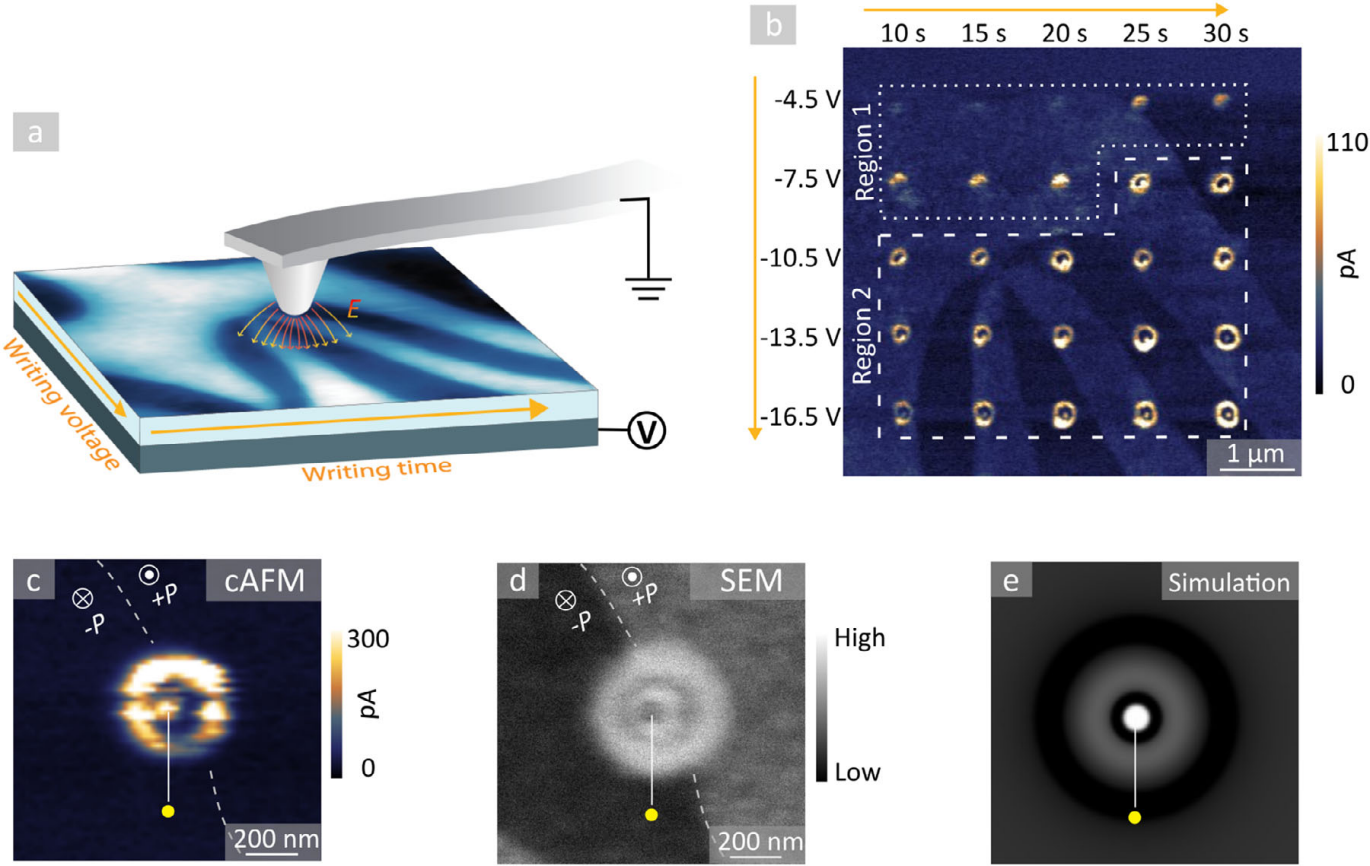
Controlling conductance by electric‐field‐induced oxygen defects. a) Illustration of the AFM‐based setup used for oxygen defect writing with orange arrows giving the directions in which the writing parameters (*U* ^write^, *t* ^write^) in (b) are varied. Negative bias voltages *U* ^write^ are applied to the back electrode. Red/yellow field lines represent the local electric field, *E*, generated by the tip. b) cAFM map showing conducting features written in Er(Mn,Ti)O_3_ on a [001]‐oriented surface using different writing parameters (*U* ^write^, *t* ^write^). The image is recorded with a positive voltage *U* ^read^ = +15 V applied to the back electrode, using the same tip as for the defect writing (Diamond coated tip, DCP 20, radius: 100 nm, K‐tek Nanotechnology, US). c) cAFM image of an extended conducting dot‐ring structure (*U^write^* = −25.5V, *t^write^* = 90 s). White symbols indicate the polarization direction, *P*, of ferroelectric domains in the background. The white dashed line indicates the domain wall position. d) SEM image of the same dot‐ring structure as in (c), captured at 500 V with 0.1 nA electron beam current and 1500 V beam deceleration using the through‐the‐lens detector (TLD). e) Simulated electric‐field‐driven distribution of oppositely charged defects (adapted with permission from ref. [27]. Copyright 2020, Springer Nature). Dark and bright contrasts indicate low and high defect concentrations, respectively. The yellow dot in (c–e) marks the location of the conducting center of the dot‐ring structures.

We begin with a systematic investigation of changes in the local transport behavior of Er(Mn,Ti)O_3_ in response to applied d.c. voltages, varying the magnitude and exposure time. To apply the voltage with nanoscale spatial precision and subsequently record conductance maps, a standard conductive atomic force microscopy (cAFM) setup is used as sketched in **Figure** **1**a, following the same protocol as in ref. [27]. To control the local transport behavior, a conducting AFM tip is brought in contact with the surface and a negative write voltage (*U* ^write^) is applied to the back of the sample (≈ 0.5 mm thick, mounted with silver paste on a metal plate) for a certain time (*t* ^write^), whereas read‐out is realized using positive voltage (*U* ^read^). Figure 1b displays a representative conductance map gained on a [001]‐oriented surface of Er(Mn,Ti)O_3_ (*U* ^read^ = +15 V) after writing a set of conducting features with varying *U* ^write^ (−4.5 to −16.5 V) and *t* ^write^ (10 to 30 s). Positions where the writing voltage was applied exhibit bright contrast, indicating an enhancement in conductance by up to one order of magnitude relative to the background. Smaller variations in the background signal *(∆I* ^domain^ ∼ 15 pA) are associated with the ferroelectric domains.^[^ ^33^, ^41^ ^]^

On a closer inspection of the conductance map in Figure 1b, we find two characteristic regions. This observation is consistent with previous work on anti‐Frenkel defects,^[^ ^27^ ^]^ where a similar experiment with varying electric‐field‐exposure time was conducted to demonstrate that they are composed of positively and negatively charged point defects. Key properties of the evolving conducting features, including their formation dynamics, atomic‐scale structure, chemical composition, and stability remain to be investigated. Conducting features written with lower (*U* ^write^, *t* ^write^)‐values tend to exhibit a dot‐like shape, as seen in the area marked with the white dotted line (Region 1). As discussed elsewhere,^[^ ^27^ ^]^ the enhanced conductance in this region originates from electric‐field‐written anti‐Frenkel defects. In contrast, for larger (*U* ^write^, *t* ^write^)‐values, a more complex pattern appears (Region 2), consisting of a conducting central dot and a conducting outer ring, separated by an intermediary region of higher resistivity. To understand the physical properties and potential functionalities associated with these dot‐ring structures, we conduct additional microscopy experiments.

Figure 1c displays a conductive atomic force microscopy (cAFM) image of an extended dot‐ring structure, which was achieved by setting the write time to *t* ^write^ = 90 s (*U* ^write^ = −25.5 V). To gain further insight into the electronic structure, we perform complementary scanning electron microscopy (SEM) measurements on the same feature, allowing for contact‐free conductance mapping.^[^ ^42^, ^43^ ^]^ Figure 1d is an SEM image recorded at the same position as the cAFM map in Figure 1c, revealing a qualitatively similar dot‐ring feature. The observation of the same patterns in cAFM and SEM is important, as it proves that the changes in conduction are intrinsic to the sample and not related to the specifics of the tip‐sample contact that co‐determines the cAFM conductance map.^[^ ^33^ ^]^ Compared to the cAFM data in Figure 1c, however, the contrast in SEM is inverted, i.e., bright conducting regions in cAFM are dark in SEM and vice versa. The same inversion effect is observed for the domain‐related contrast in the background, which can be related to the SEM imaging voltage.^[^ ^44^, ^45^ ^]^

Independent of the inverted contrast, Figure 1d unveils the existence of an extra outer ring that is not resolved by cAFM. Similar to the insulating intermediary region that separates the two conducting rings, this outer ring is brighter than the domains in the background in the SEM image, indicating that it represents a region with reduced conductance. The SEM data is remarkable as it is in one‐to‐one agreement with the transport behavior expected based on previously published numerical simulations.^[^ ^27^ ^]^ With this, the SEM results represent an important piece of the puzzle, corroborating the existence of the predicted outer region with reduced conductance, which is not resolved by cAFM. The data thus verifies the general applicability of the model used in ref. [27] to rationalize the electric‐field‐induced changes, establishing the emergence of multiple regions with higher or lower conductance than the surrounding unmodified bulk. For a direct comparison, we show the simulated distribution of $V_{O}^{\cdot \cdot }$ (oxygen vacancies with double positive charge) and $O_{i}^{\prime \prime }$ (oxygen interstitials, with double negative charge) in Figure 1e (adapted from ref. [27]). The simulation, originally published in ref. [27], assumes that $V_{O}^{\cdot \cdot }$ and $O_{i}^{\prime \prime }$ are created by the electric field under the AFM tip; they move in opposite directions due to their charge, including the recombination of $V_{O}^{\cdot \cdot }$ and $O_{i}^{\prime \prime }$. Based on the comparison of the SEM data and the simulation, we conclude that the extra outer ring resolved in Figure 1d relates to a gradient in defect concentration. This gradient results from the migration of $O_{i}^{\prime \prime }$ toward the high‐field region in the center, leading to a locally reduced defect concentration and, hence, lower conductance. In summary, Figure 1 closes the previous gap between experimental observations and numerical results and, most importantly for this work, demonstrates that by an adequate choice of the writing parameters *U* ^write^ and *t* ^write^, the local concentration of oxygen defects can be controlled. The latter allows for controlled splitting of the initially injected anti‐Frenkel pairs ($V_{O}^{\cdot \cdot },O_{i}^{\prime \prime }$)^[^ ^27^ ^]^ and a redistribution of $O_{i}^{\prime \prime }$ (i.e., the predominant type of oxygen defect in as‐grown hexagonal manganites^[^ ^28^ ^]^), giving rise to intriguing dot‐ring structures with distinct transport properties.

## 3 Microscopic Defect Formation Mechanism

**Figure 2:**
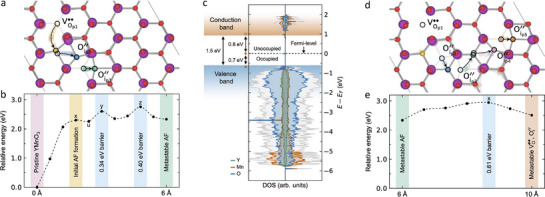
DFT simulations of the oxygen‐defect formation. a) Initial formation pathway for an anti‐Frenkel defect and (b) its energetics. Local energy maxima along the migration path are labeled x, y, and z. The unstable anti‐Frenkel configuration with $O_{i}^{\prime \prime }$ at the vacancy edge is labeled “u”. c) Electronic density of states of YMnO_3_ with the metastable (6 Å) anti‐Frenkel defect configuration. The double $O_{i}^{\prime \prime }$ configuration results in an occupied impurity state in the middle of the band gap. d) Further migration pathway for the anti‐Frenkel defect and e) its energetics. The associated migration barrier, x, is substantially lower than that for the initial defect formation and consistent with that reported for a single $O_{i}^{\prime \prime }$ migrating in bulk.^[^ ^26^ ^]^

Interestingly, the written dot‐ring structures in Figure 1 have a diameter of up to 310 nm, which is more than ten times larger than the diameter of the contact area between the tip and the sample during the defect writing process (≈ 20 nm, Note S1, Supporting Information). To analyze the defect dynamics and understand the underlying microscopic mechanisms, we perform density functional theory (DFT) calculations using hexagonal YMnO_3_ as our model system (YMnO_3_ is structurally and electronically comparable to Er(Mn,Ti)O_3_ and the absence of 4f‐electrons simplifies the DFT modelling). Calculations are done for a 3 × 3 × 1 model structure of hexagonal YMnO_3_ using the Climbing‐Image Nudged‐Elastic‐Band method for migration path modeling. **Figure** **2** displays the formation process of an anti‐Frenkel defect and the subsequent migration of oxygen interstitials and vacancies. The defect formation process considered in Figure 2a,b corresponds to a single planar oxygen atom (O_p1_, yellow) jumping to an out‐of‐plane interstitial position, which leads to a vacancy, $V_{O_{p1}}$, and a small displacement of the nearby planar oxygen (O_p2_, blue). As a consequence, a defect configuration with two off‐lattice oxygens $O_{i}^{\prime \prime }$(yellow $O_{i_{p1}}^{\prime \prime }$ and blue $O_{i_{p2}}^{\prime \prime }$) arises. Such a configuration with two $O_{i}^{\prime \prime }$ at the vacancy edge (marked “u” in the energy profile in Figure 2b) is weakly metastable with an energy barrier of 0.04 eV protecting it from collapsing upon perturbations. A further displacement of O_p1_ will cause it to reach the initial planar position of O_p2_. Meanwhile, O_p2_ has nudged another planar oxygen off its lattice site and pushed this third planar oxygen ($O_{i_{p3}}^{\prime \prime }$, green) to a similar interstitial position, creating a new double $O_{i}^{\prime \prime }$ configuration farther away (≈ 6 Å) from the vacancy. We consider this the first metastable anti‐Frenkel defect configuration. Note that the defect configuration with two interstitial $O_{i}^{\prime \prime }$ is different from the common view of anti‐Frenkel defects, which refers to only one vacancy and one interstitial $O_{i}^{\prime \prime }$. We observe the double $O_{i}^{\prime \prime }$ configuration by starting from a single interstitial $O_{i}^{\prime \prime }$ and running molecular dynamics at 300 K, where the system turns fast to the energetically more favorable double $O_{i}^{\prime \prime }$ configuration. At elevated temperature, i.e., at 1000 K, recombination takes places within a few picoseconds, following the reverse trajectory of the zero‐Kelvin‐optimized migration path in Figure 2a.

Figure 2b shows that the initial vacancy creation step (labeled x, yellow) requires 2.30 eV of energy and that there is only a subtle barrier of 0.04 eV backward. Further migration toward the first metastable anti‐Frenkel configuration (green) has two modest barriers of 0.34 eV (labeled y, blue) and 0.40 eV (labeled z, blue). Concerning the stability, the first migration barrier backward, z, is 0.43 eV. The corresponding electronic density of states (DOS, Figure 2c) shows an impurity state of one electron in the middle of the band gap, which is not present in the defect‐free system (Figure S1, Supporting Information) and promotes enhanced electronic hopping conductivity.^[^ ^27^ ^]^

We find that the stability of the defect complex gets further enhanced as oxygen interstitials and vacancies move away from each other. One possible pathway is illustrated in Figure 2d, showing how O_p2_ and O_p3_ acquire new on‐lattice sites by pushing two other oxygen atoms into a double $O_{i}^{\prime \prime }$ configuration (pink $O_{i_{p4}}^{\prime \prime }$ and orange $O_{i_{p5}}^{\prime \prime }$). In this process, which has a migration barrier of 0.61 eV (labeled x, blue in Figure 2e), the distance between oxygen interstitials and vacancy grows to 10 Å. A second migration pathway is presented in Note S2 and Figure S1 (Supporting Information), leading to an energetically less favorable anti‐Frenkel defect configuration at 9 Å separation. However, repeating the migration step once more in the same direction (12 Å separation) reduces the energy by 0.44 eV with respect to the previous configuration, indicating improved stability as the distance between double $O_{i}^{\prime \prime }$ and $V_{O_{p1}}$ gets larger (not shown). The reduction in total energy at larger distances does not mean repulsion of the charged species per se, but the effect is related to effective charge screening around $V_{O}^{\cdot \cdot }$ and $O_{i}^{\prime \prime }$, as indicated by the local Bader charges,^[^ ^27^ ^]^ and reduced strain overlap around the defects.

In summary, our calculations show two important aspects, that is, i) the system can reduce its energy by separating the oxygen interstitials and vacancies of electric‐field‐induced anti‐Frenkel defects at larger distances, and ii) there are energy barriers for recombination that lead to different metastable configurations once the driving electric field is switched off. The model indicates a remarkably high mobility of oxygen defects in hexagonal manganites, which is consistent with previous studies^[^ ^28^, ^46^ ^]^ and the large migration distances we observe experimentally (Figure 1). Most importantly for this work, the proposed microscopic mechanism, i.e., the electric‐field‐driven splitting of anti‐Frenkel defects, implies that the formation of the dot‐ring structures leads to spatially separated $V_{O}^{\cdot \cdot }$‐rich and $O_{i}^{\prime \prime }$‐rich areas, corresponding to electron‐ and hole‐doped regions, respectively.

## 4 High‐Resolution Imaging of Vacancy‐ and Interstitial‐Rich Regions

**Figure 3:**
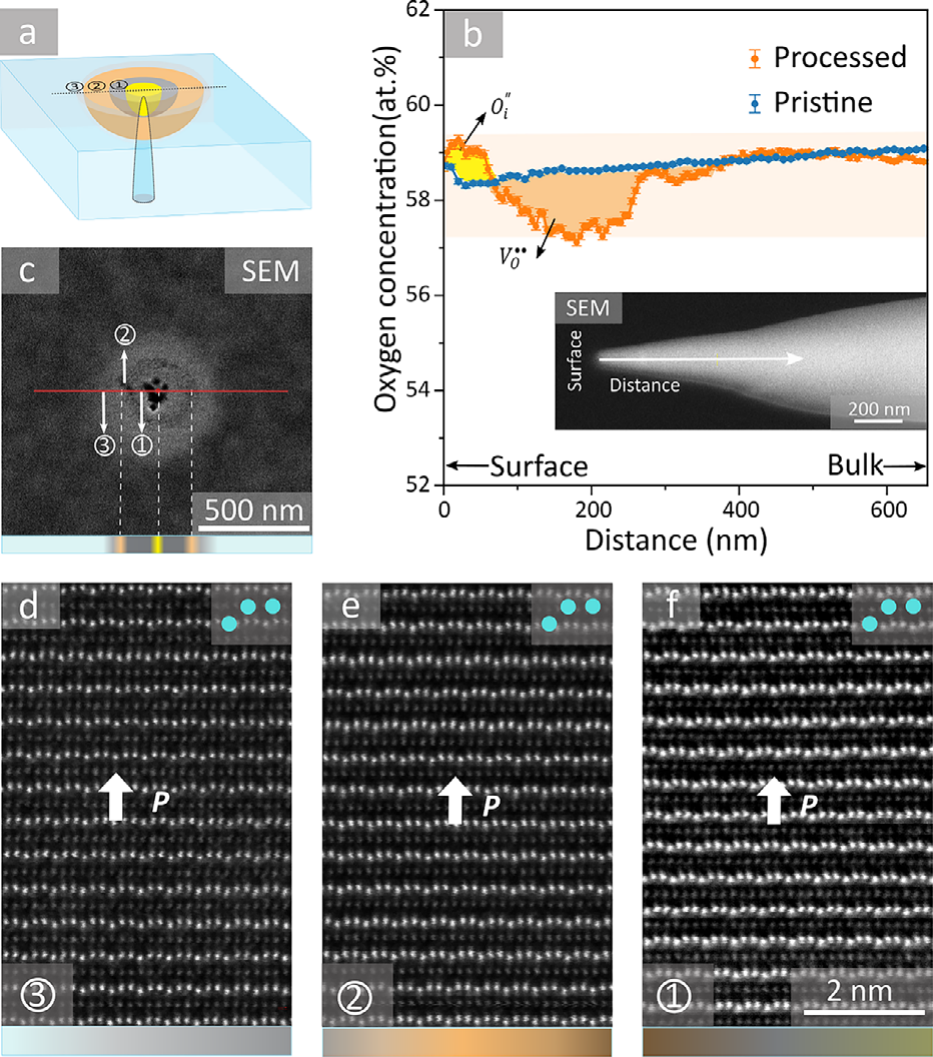
APT and STEM analysis of the dot‐ring structure. a) Illustration of the region from which a needle for APT‐based chemical analysis is prepared. Colors and numbers indicate the characteristic regions we resolve by cAFM and SEM (yellow = conducting inner dot; grey = recombination region ①; orange = outer conducting ring ②; light blue = bulk ③. b) Oxygen concentration profile measured by APT for an electric‐field written dot‐ring structure (processed) and an Er(Mn,Ti)O_3_ reference sample (pristine). The $O_{i}^{\prime \prime }$‐rich region (yellow) corresponds to an accumulation region of interstitial oxygen ions (−2 charge) which, hence, is negatively charged with respect to the bulk. In contrast, the $V_{O}^{\cdot \cdot }$‐rich region (orange) is positively charged due to enhanced density of oxygen vacancies (+2 charge). The APT needle extracted from the processed region is displayed in the inset. c) SEM image of another dot‐ring structure written for subsequent structural analysis (*U* ^write^= −22.5V and *t* ^write^= 60 s), captured under an electron beam of 1.25 kV, 0.1 nA, with the TLD detector. The red line marks the location from which a cross‐sectional lamella is extracted for HAADF‐STEM imaging. d),e),f) HAADF‐STEM data for regions ①, ②, and ③.

To experimentally verify the emergence of such $V_{O}^{\cdot \cdot }$‐rich and $O_{i}^{\prime \prime }$‐rich regions, we conduct atom probe tomography (APT) measurements. For this purpose, needle‐shaped specimens are extracted from two samples using a focused ion beam (FIB) as sketched in **Figure** **3**a. The first specimen (processed) is taken from an area with an electric‐field written conducting dot‐ring structure (*U* ^write^ = −52.5 V, *t* ^write^ = 60 s), as sketched in Figure 3a. The second specimen (pristine) is from an Er(Mn,Ti)O_3_ reference sample, which is cut from the same crystal and prepared under identical conditions as the processed sample (see Methods for details of the sample preparation and Figure S2, Supporting Information for APT mass spectra).

The needle‐like shape enables the high electric fields required for field evaporation of surface atoms in APT as introduced, for example, in ref. [47]. Figure 3b compares the respective oxygen concentration profiles, which are derived by integrating the 3D APT data perpendicular to the axis of the needle‐shaped specimens (see inset to Figure 3b). We note that a 3.95% offset is added to the Er(Mn,Ti)O_3_ reference data to align the curves in the bulk region, accounting for variations in the charge‐state ratio (CSR), as discussed in ref. [48] (see Figures S3 and S4, Supporting Information for details). The comparison shows a pronounced variation in oxygen concentration in the electrically modified sample, which is absent in the pristine state. In the electrically modified region, a substantial enhancement in oxygen concentration is measured near the surface (yellow), followed by a region with reduced oxygen concentration (orange). This oxygen concentration profile is consistent with our model and the numerical simulations, and it confirms that the conducting center of the dot‐ring structures corresponds to an $O_{i}^{\prime \prime }$‐rich region, whereas the conducting ring coincides with a $V_{O}^{\cdot \cdot }$‐rich region. However, the width of the intermediary recombination regime obtained based on the APT concentration profile (measured parallel to the [001]‐direction) is much smaller than in the cAFM and SEM data (measured perpendicular to the [001]‐direction). A possible explanation for this anisotropy is the difference in ionic mobility along and perpendicular to the [001]‐axis in hexagonal manganites.^[^ ^28^, ^38^, ^49^ ^]^ Furthermore, the APT data reflect that the volume fractions of the oxygen‐deficient (orange) and ‐rich (yellow) regions are different, which we attribute to field‐driven $O_{i}^{\prime \prime }$‐migration and partial desorption of oxygen ions at the sample surface during the writing of the dot‐ring structures, similar to other oxide systems, ^[^ ^50^, ^51^ ^]^ and/or loss of material at the surface during sample preparation. Importantly for this work, together with the transport characteristics measured at the sample surface (Figure 1c,d), the APT results establish the obtained dot‐ring structure as a specific type of electric‐field‐induced defect complex, with unique electronic properties and characteristic chemical structure, that is fundamentally different from the initial defect state where $V_{O}^{\cdot \cdot }$ and $O_{i}^{\prime \prime }$ exist in pairs.

Representative structural data for the characteristic regions ① to ③ as defined for the dot‐ring structure in Figure 3c are displayed in Figure 3d–f. The SEM data shows qualitatively the same intensity distribution as in Figure 1d, aside from an additional darker spot in the center where the tip was placed during defect writing, indicating that the high electric field locally altered the sample (Figure S5, Supporting Information). A color bar below the SEM image identifies the different regions of the dot‐ring structure, analogous to the illustration in Figure 3a. The high‐angle annular dark‐field scanning transmission electron microscopy (HAADF‐STEM) data from regions ① to ③ is gained from a cross‐sectional lamella extracted at the position marked by the red line in Figure 3c using FIB (see Figure S5, Supporting Information for details). The HAADF‐STEM scans view down the [$\bar{1}00$] direction and exhibit the characteristic down‐up‐up pattern of Er atoms separated by layers of Mn atoms, reflecting the structural integrity after splitting the anti‐Frenkel pairs into $V_{O}^{\cdot \cdot }$‐rich and $O_{i}^{\prime \prime }$‐rich regions. We note that we do not resolve a signature of these defect‐rich regions by electron energy loss spectroscopy at room‐temperature, which may be attributed to electron‐beam induced changes,^[^ ^52^ ^]^ further promoted by a much higher defect mobility compared to the cryogenic temperature at which the APT data in Figure 3 was acquired (T = 25 K). Importantly for this work, despite the pronounced variation in conductance measurable in cAFM and SEM, the HAADF‐STEM data show qualitatively the same Er displacement within the three regions (down‐up‐up = +*P*). This finding leads us to the conclusion that the redistribution of oxygen defects is the main driving force for the emergence of the conducting dot‐ring structures, whereas potential polarization‐dependent contributions can be excluded.

## 5 Transient Nature of Functionalized Regions

**Figure 4:**
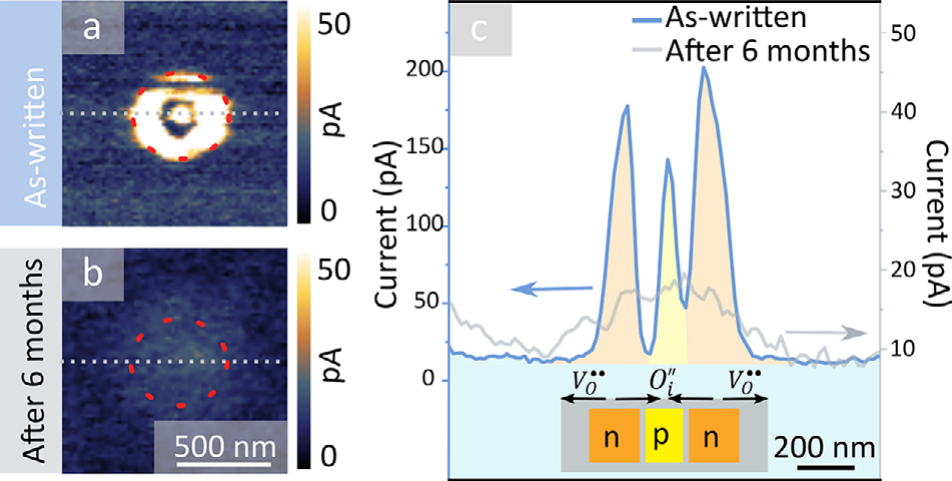
Temporal stability of electric‐field written dot‐ring structures. a) cAFM image showing the as‐written state of a conducting dot‐ring structure. b) cAFM image captured at the same position as in (a) six months after writing the defect structure. c) Current profiles comparing the as‐written and the six‐months‐aged defect structures. The illustration at the bottom depicts the distribution of $O_{i}^{\prime \prime }$ and $V_{O}^{\cdot \cdot }$ associated with the as‐written defect structure, transiently generating p‐ and n‐type regimes, comparable to a dipolar npn‐junction.

In contrast to the anti‐Frenkel defects that arise for lower (*U* ^write^, *t* ^write^)‐values (Figure 1b, region 1), the $V_{O}^{\cdot \cdot }$‐rich and $O_{i}^{\prime \prime }$‐rich regions are no longer charge‐neutral. As a consequence, different stability criteria are expected to apply in the latter case, which we investigate in **Figure** **4**. Figure 4a displays a cAFM image gained immediately after writing a conducting dot‐ring structure with up to 10 times higher conductance than the background (*U* ^write^ = −21.0 V, *t* ^write^ = 90 s). After writing such dot‐ring structures, they maintain their initial diameter (here, ≈ 540 nm, red dashed line) and transport behavior with no substantial deviations on the time scale of days. The SEM image in Figure 1d, for example, was captured six days after the cAFM image in Figure 1c. On the timescale of months, however, we observe substantial changes. By performing a cAFM scan six months after the initial scan at the same position (Figure 4a), we find that the well‐defined dot‐ring structure has vanished. Instead, we now observe a rather blurred spot of only about two to three times higher conductance than the surrounding bulk (Figure 4b). For a quantitative comparison, line plots extracted along the white dashed lines in the cAFM images in Figure 4a,b are presented in Figure 4c, highlighting the evolution from a conducting dot‐ring structure with three well‐defined maxima to a broader region of slightly elevated conductance. The time‐dependent behavior of the $V_{O}^{\cdot \cdot }$‐rich and $O_{i}^{\prime \prime }$‐rich regions are thus very different from regions with electric‐field‐induced anti‐Frenkel defects, which were reported to exhibit robust enhanced transport properties even after 24 months.^[^ ^27^ ^]^

The substantially lower stability compared to the anti‐Frenkel defects can be understood based on the positive and negative charges carried by the $V_{O}^{\cdot \cdot }$ and $O_{i}^{\prime \prime }$, respectively. Because of the charge of the oxygen defects, the gradients in defect concentration, as observed in Figure 3b, lead to potential gradients and built‐in electric fields that promote the diffusion of charged oxygen defects. Conceptually, the effect is the same as reported in ref. [53, 54], where it was observed that oxygen defects collected by a biased tip diffuse and redistribute over time. As first tests suggest, such temporal diffusion and recombination processes may further be promoted by electric fields of inverted polarity, giving additional control possibilities for future studies (Figure S6, Supporting Information).

## 6 Outlook

The demonstrated electric‐field control of oxygen defects gives new opportunities for the functionalization of oxide semiconductors. Because of the different types of oxygen defects that accumulate at the center ($O_{i}^{\prime \prime }$) and in the ring ($V_{O}^{\cdot \cdot }$) of the dot‐ring structures studied in this work, different electronic properties arise than in the surrounding material. In particular, $O_{i}^{\prime \prime }$ acts as an acceptor and promotes p‐type conductivity, whereas $V_{O}^{\cdot \cdot }$ acts as a donor, giving rise to n‐type conductivity. Thus, the electric‐field‐induced defect arrangement can be considered as a bipolar npn‐junction as sketched in Figure 4c. Bipolar npn‐junctions are basic electronic components used for amplification and switching,^[^ ^55^ ^]^ enabling the control of currents flowing from the collector to the emitter via a small current injected at the base.

The electric‐field‐driven splitting of anion interstitial‐vacancy pairs – or defect complexes comprised of charged ions and/or vacancies in general – represents a promising pathway to functionalize oxide systems, simultaneously controlling the distribution of both p‐ and n‐type carriers with nanoscale spatial precision. Similar effects are expected for oxides which offer both structural openness and high redox flexibility. Candidate systems include other members from the hexagonal manganites family,^[^ ^56^ ^]^ fluorite‐type oxides,^[^ ^57^ ^]^ as well as perovskite‐derived structures, such as Ruddlesden‐Popper oxides,^[^ ^58^ ^]^ Silleń oxychlorides,^[^ ^59^ ^]^ and Brownmillerite oxides,^[^ ^60^ ^]^ where oxygen defects can move relatively freely compared to standard, more densely packed, perovskite systems. The npn‐like arrangement discussed in this work degrades on the timescale of a few months, implying that the system is functionalized only temporarily. Such temporary behavior is specifically wanted in transient electronics, where the functionalization is required to disappear over time,^[^ ^22^ ^]^ giving additional application opportunities for oxide materials.

Now that we have shown that interstitial‐rich (p‐type) and vacancy‐rich (n‐type) regions are formed under applied d.c. voltage, the next step is to test and utilize the written structures in device‐relevant geometries. One of the key challenges will be to establish ohmic electrical contacts to the modified region, which is crucial to avoid the formation of an additional barrier at the electrode‐surface contact. The ohmic contacts will allow for operation at reduced voltage, mitigating the risk of unwanted electric‐field‐driven modifications of the dot‐ring structures. In conclusion, the results foreshadow conceptually new application opportunities that are of interest for the development of transient oxide nanoelectronics, leveraging the volatile nature of the electric‐field‐induced nano‐regions to locally functionalize a system for a programmed period of time.

## 7 Experimental Section

### Sample Preparation

Er(Mn,Ti)O_3_ single crystals were grown using the pressurized floating‐zone method.^[^ ^61^ ^]^ The samples were then oriented by Laue diffraction and cut into 1 mm‐thick pieces with polarization directions perpendicular to the sample surface, yielding specimens with out‐of‐plane polarization. Following this, the samples were lapped with an Al_2_O_3_ (9 µm grain size) fluid and polished with silica suspension (Logitech, SF1 polishing suspension) to achieve a smooth surface. After polishing, all samples were annealed in an Entech tube furnace under an N_2_ or 5% H_2_/N_2_ atmosphere at 300 °C for 48 h, with a heating and cooling rate of 5°C per minute, following the same procedure as in ref. [25].

### Scanning Probe and Scanning Electron Microscopy

cAFM measurements and defect writing were carried out using a Cypher ES environmental AFM (Oxford instruments) equipped with diamond‐coated AFM probe tips (DCP 20). Bias voltages were applied to the sample back‐electrode while the tip was grounded. SEM imaging was performed with a Thermo Fisher Scientific G4UX Dual‐beam FIB‐SEM operated in secondary electron mode.

### Scanning Transmission Electron Microscopy (STEM)

The lamella for STEM measurements was prepared using Thermo Fisher Scientific G4UX Dual‐beam FIB‐SEM. A standard extraction procedure was conducted^[^ ^62^ ^]^ and the milling processes on both sides of the lamella was guided by markers (Figure S5, Supporting Information). Low‐energy ion beam polishing (2 kV, 0.11 nA) was applied to minimize the beam‐induced damage.^[^ ^63^ ^]^ STEM imaging was performed using a JEOL JEM‐ARM200F microscope. The probe size was set to 8C with a beam‐limiting aperture of 40 µm, optimized for high‐resolution STEM images. A camera length of 4 cm was used for detection, which helps distinguish heavier atoms in the material through the HAADF detector. This detector is sensitive to Rutherford scattered electrons, and at this camera length, the collection angle starts at 550 mrad, providing enhanced contrast, particularly at the Er sites.

### Atom Probe Tomography (APT)

APT needles were prepared using a Thermo Fisher Scientific G4UX Dual‐beam FIB‐SEM. The dot‐ring structure was marked by an electron beam deposited carbon (C) marker and followed the extraction procedure described in ref. [64]. APT data were acquired using a Cameca LEAP 5000XS, operating in laser pulsing mode using 30 pJ pulse energy at 250 kHz pulsing frequency. The specimen temperature was set to 25 K, and the detection rate was set to 0.5% (1 atom detected every 200 pulse on average). Data reconstruction was performed with Cameca APSuite 6, using the dynamic voltage reconstruction method,^[^ ^65^ ^]^ with the initial tip radius determined by SEM and the reconstruction parameters set to achieve correct interatomic plane distances along the 001‐pole.

### Density Functional Theory (DFT) Calculations

Detailed information about the DFT calculations, including computational parameters and methods, is provided in Note S2 (Supporting Information).

## Conflict of Interest

The authors declare no conflict of interest.

## Author Contributions

J.H. performed the SPM and SEM experiments, prepared the lamellas for STEM using FIB, assisted with the sample preparation for APT, and analyzed the data. U.L. and P.E.V. carried out the STEM measurements and performed relevant data analysis. K.A.H. prepared the APT needle using FIB, conducted the APT measurements, and analyzed the corresponding data. The work by J.H., U.L., and K.A.H. was done under supervision by D.M.; D.R.S., J.A., D.G., and S.M.S. performed DFT calculations and analyzed the data. K.S. conducted the numerical simulation of the electric‐field‐driven redistribution of defects. D.M.E and E.D.R contributed to discussions on experimental processes. Z.Y. and E.B. provided the materials. D.M. devised and coordinated the project. J.H. and D.M. wrote the manuscript, with support from D.R.S., J.A., and S.M.S. for the DFT‐related sections. All authors discussed the results and contributed to the final version of the manuscript.

## Supporting information



Supporting Information

## Data Availability

The data that support the findings of this study are available from the corresponding author upon reasonable request.
