# Supplementary material for: Local p‐ and n‐Type Doping of an Oxide Semiconductor via Electric‐Field‐Driven Defect Migration
Source: Adv Sci (Weinh). 2025 Sep 18;12(43):e06629. doi: 10.1002/advs.202506629 (PMC12631835; doi:10.1002/advs.202506629)
Supplement: Supplementary file 1 — Supporting Information [file ADVS-12-e06629-s001.pdf]

Supporting Information

Local p- and n-type doping of an oxide semiconductor via electric-field-driven defect migration

Jiali He[1], Ursula Ludacka[1], Kasper A. Hunnestad[1,2], Didrik R. Småbråten[1,3], Konstantin Shapovalov[4], Per Erik Vullum[5,8], Constantinos Hatzoglou[1], Donald M. Evans[1,3], Erik D. Roede[1], Zewu Yan[6,7], Edith Bourret[7], Sverre M. Selbach[1], David Gao[8,9], Jaakko Akola[8,10] and Dennis Meier[1,11]*

[1]Department of Materials Science and Engineering, NTNU Norwegian University of Science and Technology, 7034 Trondheim, Norway.

[2]Department of Electronic Systems, NTNU Norwegian University of Science and Technology, 7034 Trondheim, Norway.

[3]Department of Sustainable Energy Technology, SINTEF Industry, 0373 Oslo, Norway.

[4]Theoretical Materials Physics, Q-MAT, University of Liège, B-4000 Liège, Belgium.

[5]SINTEF Industry, 7034 Trondheim, Norway

[6]Department of Physics, ETH Zürich, 8093 Zürich, Switzerland.

[7]Materials Sciences Division, Lawrence Berkeley National Laboratory, Berkeley, CA 94720, USA.

[8]Department of Physics, NTNU Norwegian University of Science and Technology, 7491 Trondheim, Norway.

[9]Nanolayers Research Computing, London, NW9 6PL, UK.

[10]Computational Physics Laboratory, Tampere University, FI-33014 Tampere, Finland.

[11]Faculty of Physics, University of Duisburg-Essen, 47057 Duisburg, Germany

Supplementary Notes

Supplementary Note 1: Tip-sample contact

In the Hertz contact model, the probe tip is approximated by a sphere that is in contact with the surface. The radius (a) of the contact area between the tip and sample is

$$a^3 = \frac{3FR}{4E^*},$$

where F represents the applied force, R denotes the tip radius, and E^* is the effective elasticity modulus.

E^* can be calculated by [1]

$$\frac{1}{E^*} = \frac{1-\nu_1^2}{E_1} + \frac{1-\nu_2^2}{E_2},$$

and E_1, E_2 are the elastic moduli and ν_1, ν_2 the Poisson's ratios.

The value of E^* is determined to be 119 GPa, based on setting Young's modulus to 220 GPa and Poisson's ratio to 0.277 [2] for both the tip and the sample. For DCP 20 probe tips with a curvature radius of 100 nm, the spring constant was calibrated as 92.68 nN/nm, with a deflection inverse optical lever sensitivity (InvOLS) of 17.73 nm/V and a setpoint of 1V applied. Based on these parameters, the diameter of the contact area is calculated to be ≈20 nm.

Supplementary Note 2: DFT simulation details and alternative defect migration path

The electronic structure simulations of the defect migration were performed using the CP2K and VASP program packages [3], applying a combination of the PBE and range-separated PBE0 functionals for the exchange-correlation energy [4],[5]. CP2K uses a dual Gaussian–plane wave basis set where the kinetic energy cut-off for the auxiliary plane waves for electron density has been set 600 Ry [6]. A molecularly optimized double- ζ valence-polarized (DZVP) Gaussian basis [7] was used for all elements with valence configurations Y (4s, 5s, 4p, 4d), Mn (3s, 4s, 3p, 3d) and O (2s, 2p) and together with the Goedecker-Teter-Hutter (GTH) pseudopotentials [8]. The Hartree-Fock exchange component in the hybrid PBE0 functional improves the description of electronic structure for transition metal oxides, and unlike the traditional LDA+U method, PBE0 does not require tuning parameters. Our migration calculations employ a tested approach [9] where PBE0 is used for the 3d-orbitals of Mn (in the spirit of the +U correction) while the rest are treated with PBE. This selection, together with the fact that the computational cost of the Hartree-Fock exchange is reduced by using the auxiliary density-matrix method (ADMM) [10] makes the DFT simulations efficient.

The geometry optimizations were performed by the Broyden-Fletcher-Goldfarb-Shanno (BFGS) algorithm for individual structures and the nudged-elastic-band method (NEB) with the climbing image algorithm [11],[12] for modeling the migration paths. The 3×3×1 model structures of hexagonal $YMnO_3$ included 270 atoms with an initial antiferromagnetic ordering of local Mn magnetic moments, and periodic boundary conditions were used in all directions. An extended 3×6×1 model structure (540 atoms) where the system was replicated in one

direction was used for investigating the anti-Frenkel defect energetics at longer separations based on structural optimizations of the anti-Frenkel defect configurations. The electronic structure analysis of the representative 3×6×1 anti-Frenkel defect configuration is performed by the projector augmented wave (PAW) method as implemented in VASP [13],[14],[15] using the PBEsol+U[16] functional with U = 5 eV applied to Mn 3d states. Y (4s, 5s, 4p, 4d), Mn (3s, 4s, 3p, 3d), and O (2s, 2p) were treated as valence electrons, with a plane-wave cut-off energy of 550 eV and a k-point density of 2×1×2.

Inspection of effective (Bader) charges shows that $O_i^{''}$ have reduced negative charges of -1.13e in comparison with -1.28e in the pristine sample. The Mn atom that is bound to both $O_i^{''}$ has an increased cationic charge of +1.90 eV (1.73 in pristine), nominally referred to as Mn^{4+}. One of the three Mn that surround $V_O^{\cdot\cdot}$ has a reduced charge of +1.37e (nominally Mn^{2+}), but otherwise, there are no significant changes in charges elsewhere. The magnetic moments of Mn atoms are consistent with the above observations of charge depletion/accumulation while the initial antiferromagnetic ordering prevails. The charge compensation of the anti-Frenkel defect is comparable to our previous work on the double $O_i^{''}$ configuration [9]. Furthermore, it is consistent with the charge compensation of the constituting $O_i^{''}$ and $V_O^{\cdot\cdot}$ in bulk [17],[18], with the exception that the anti-Frenkel defect is compensated by oxidizing (reducing) one Mn^{3+} to Mn^{4+} (Mn^{2+}) compared to two Mn^{4+} (Mn^{3+}) for the isolated bulk defects due to the relative positions of the double $O_i^{''}$ and the $V_O^{\cdot\cdot}$ as discussed previously [9]. The changes in effective charges highlight that anti-Frenkel defect formation (migration of O anion) is partially counterbalanced by redistribution of the (negative) charge which reduces the attractive Coulomb interaction between the vacancy and double $O_i^{''}$ configuration.

In comparison to the anti-Frenkel defect migration path presented in the main text, an alternative migration path is displayed in Fig. S 1, and it considers the direction where double $O_i^{''}$ will have the least interaction with the vacancy and its periodic images. As above, the same concerted migration mechanism leads to a detour with two barriers and two minima. The first barrier is 0.88 eV and leads to an anti-Frenkel defect configuration at 0.30 eV (AF2 in Fig. S 1b). After the second barrier of 0.65 eV, the next anti-Frenkel defect state is achieved at 0.86 eV (AF3 in Fig. S 1b). Interestingly, the first anti-Frenkel defect configuration is farther away from the vacancy (10 Å) than the second one (9 Å), while the "dimer" is oriented in a different direction. Both configurations are higher in energy than the starting position (AF at 6 Å), *i.e.,* it costs additional energy to increase the anti-Frenkel defect separation for this process. However, by repeating the detour process once more for an extended 3×6×1 model structure (to accommodate periodic boundary conditions) in the same direction such that the final interstitial-vacancy separation increases to 12 Å reduces the total energy by 0.44 eV with respect to the previous step (9 Å). This gives indications that the situation becomes energetically more feasible at longer distances (>10 Å), as one would expect based on charge screening, and that there are (several) substantial energy barriers for recombination once the driving electric field is switched off.

A comparison of the projected density of states (DOS) of pristine $YMnO_3$ and with the metastable (6 Å) AF configuration is shown in Fig. S 1c.

Supplementary Figures

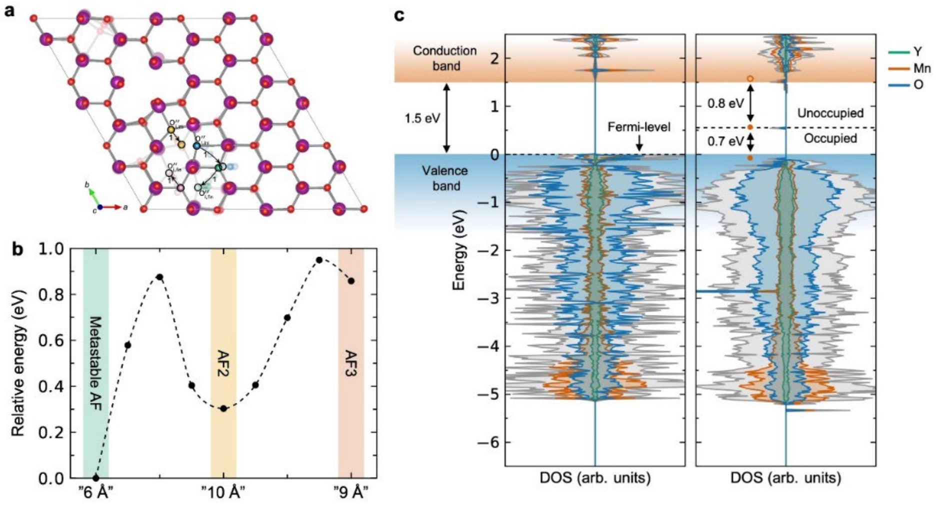

Fig. S 1 Anti-Frenkel defect migration path. a, Pathway and (**b**) its energetics. The concerted D-tour migration involves an intermediate double O_i'' configuration (blue and green, 0.30 eV) before reaching the final state (green and pink, 0.86 eV). **c**, Comparison of the electronic DOS of pristine $YMnO_3$ (left) with the metastable (6 Å) anti-Frenkel defect configuration (right). The DOS for the defect system is shifted according to the Fermi level relative to the pristine system.

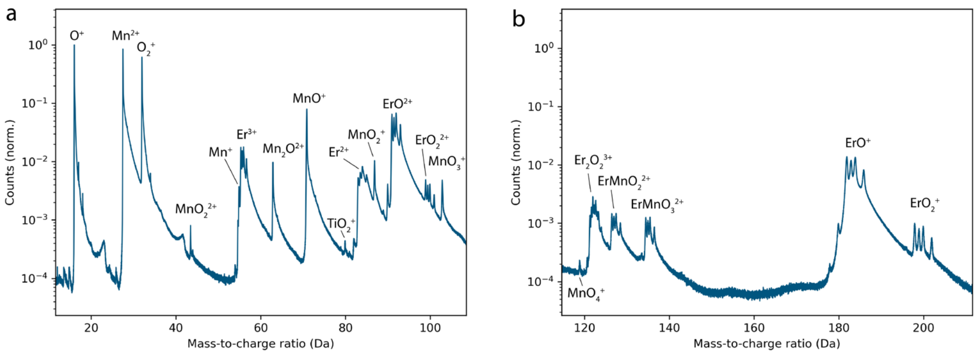

Fig. S 2 APT mass spectra. a,**b**, Mass spectra of the $Er(Mn,Ti)O_3$ specimen with electric-field written dot-ring structure analyzed in Fig. 3 of the main text. The labeled ionic species indicate the ions used for the reconstruction and the chemical analysis (only the main peaks are labeled, but all isotopes are included). The y-axis is normalized to the largest peak in the spectrum.

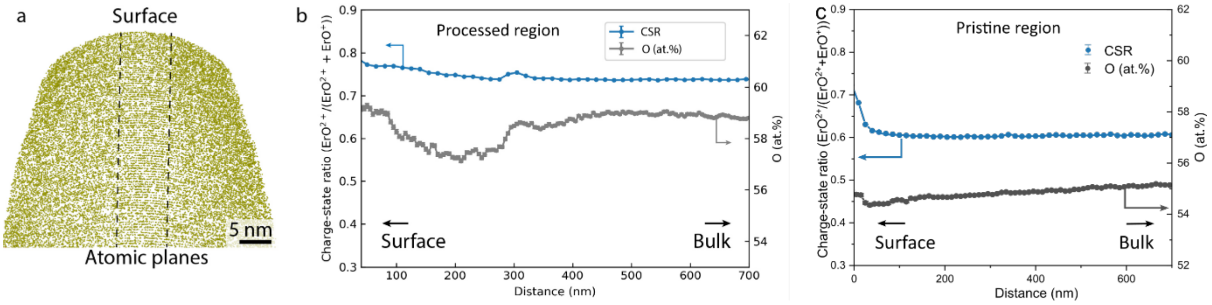

Fig. S 3 Extended APT analysis. a, Section of the APT reconstruction of the sample with electric-field written dot-ring structure analyzed in Fig. 3 in the main text. Atomic planes are visible at the 001-pole. Only the Mn ions are used for visualization. **b**, CSR profile for the processed sample, showing a slow decay over the first 400 nm before reaching a stabile value, with a small increase around 300 nm. The minor changes in the CSR (see ref. [19] for a quantitative analysis) do not directly correlate with the observed changes in the oxygen concentration. **c,** CSR profile for the reference sample (pristine). The difference in CSR for the two samples leads to an offset in the measured oxygen concentration, which was accounted for by in Fig. 3b as explained in the main text.

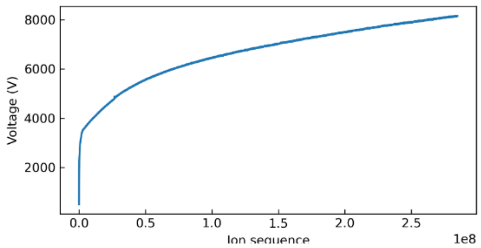

Fig. S 4 APT voltage profile. The graph shows the voltage profile used for the dynamic reconstruction method. The voltage increases gradually, indicating a continuously increasing specimen radius as expected during APT analysis. A minor spike in the voltage is observed after 30 M ions around 5 kV, which coincides with the small anomaly in CSR at 300 nm (Fig. S3) and likely corresponds to a microfracture.

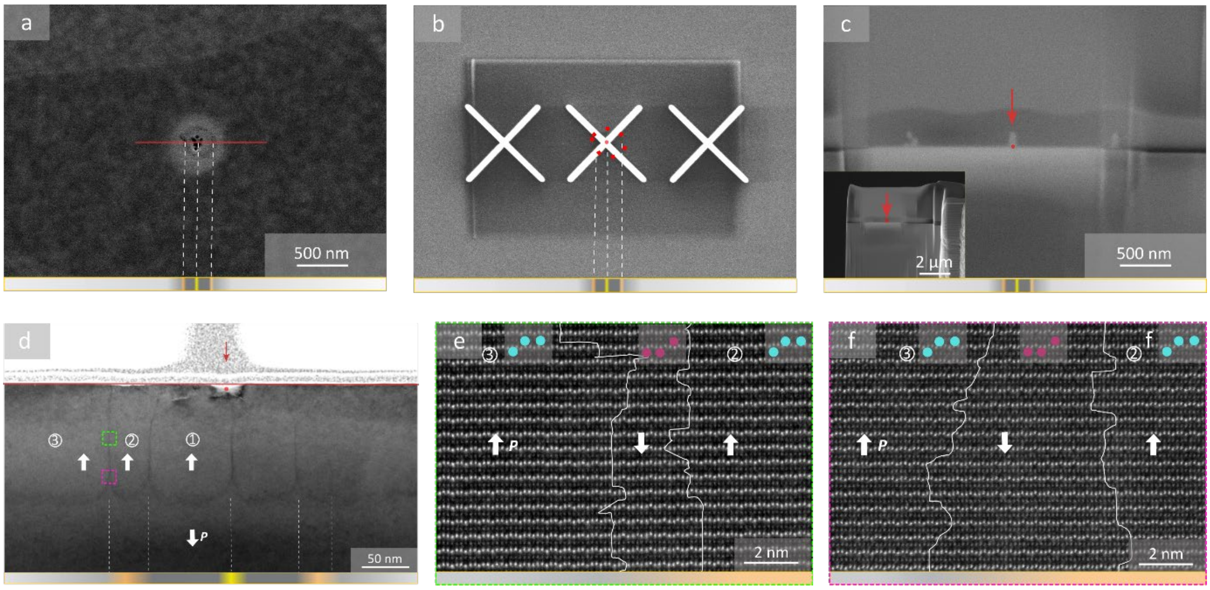

Fig. S 5 Sample preparation and STEM analysis. a, Large-scale SEM image of the dot-ring structure presented in Fig. 3c in the main text (1.25 kV, 0.1 nA, TLD). Colors below illustrate the conducting central dot (yellow) and ring (orange) as indicated by the dashed lines. **b**, SEM image of the Pt markers (white crosses; deposited using Pt precursor gas in a low electron beam current, 1.25 kV at 50 pA, in immersion mode) on the dot-ring structure (red dotted line) that were used to extract a cross-sectional lamella from the region of interest. **c**, Cross-sectional SEM image of the lamella; the bright region corresponds to the electron-transparent area [20]. Inset: SEM overview image of the final lamella (4.00 kV, 0.1 nA, ICE detector). Red arrows indicate the position of the marker centered over the dot-ring structure. **d**, Overview STEM image (200 kV, CCD); the red line corresponds to the position marked in **a**. Bright contrast at the top (red dot) indicates local structural changes on the length scale of ≈ 10 nm, which we attribute to the electric field at the position where the AFM tip was placed for defect writing (red arrow) [21]. Note that the electric fields applied during defect writing are not sufficient to electrically pole the material, and much larger voltages or cryogenic temperatures are required to irreversibly switch hexagonal manganites (for a detailed discussion see, e.g., ref.[22] and ref.[23]). The local direction of *P* is indicated by white arrows, and numbers ①, ②, and ③ label the region for which HAADF-STEM images are presented in the main text. The color bar is a guide to the eye with the same color code as in **a**-**c**. **e**,**f**, HAADF-STEM images from the regions marked by green and pink frames in **d**. **e** and **f** show that the darker lines between ①, ②, and ③ in **d** correspond to domains of opposite polarization, pointing in the same direction as in the region at the bottom of **d**. As the voltages applied for defect writing cannot switch the polarization direction, we attribute the appearance of upward-polarized domains to electron-beam induced switching[24],[25], with a preferred appearance of -P domains in transition regions where the transport behavior changes from insulating to conducting. Most importantly for this work, the data corroborates that the local transport properties do not correlate with the direction of *P*, excluding a polarization driven mechanism.

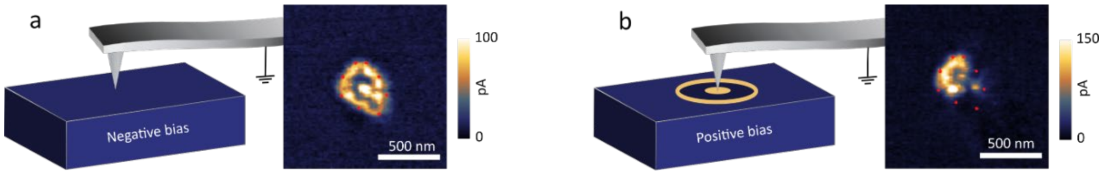

Fig. S 6 Evolution of an electric-field-written dot-ring structure under positive bias. a, Initial dot-ring structure written with U^{write} = -30 V and imaged with U^{read} = +12 V voltage, using a CDT-NCHR doped diamond tip with voltage applied to the back electrode. **b,** cAFM image of the same region as in (a) after applying a positive bias voltage (+30 V) while the tip was positioned on the inner dot. The red dotted circle in both panels outlines the original dot-ring structure. After applying positive bias, the region with enhanced conduction becomes smaller and partially vanishes. This observation suggests that the reversed electric field (+30 V) promotes defect migration in the opposite direction than the writing field (-30 V) and partial recombination of oxygen vacancies and interstitials. It is important to note, however, that by changing the direction of the electric field, the initial defect migration is not simply reversed. Instead, a more asymmetrical conducting structure arises that is more difficult to interpret. The latter may be expected as migration pathways will differ, promoted by the different environment (e.g., defect density and conduction gradients), as well as the different electric-field distribution under the tip (due to variations in surface cleanliness, humidity, conduction). Thus, although the experiment shows the general possibility to modify conducting dot-ring structures by application of positive bias voltage and partially inverting / suppressing the initial functionalization, more extended studies are required to understand and control the effect, which goes beyond the scope of this work.

References

[1] V. L. Popov, M. Heß, E. Willert, *Handbook of Contact Mechanics*, **2019**.

[2] A. Chadli, M. Halit, B. Lagoun, F. Mohamedi, S. Maabed, A. Cheriet, E. Hlil, H. Farh, *Solid State Phenom.* **2019**, *297*, 120.

[3] J. Vandevondele, M. Krack, F. Mohamed, M. Parrinello, T. Chassaing, J. Hutter, *Comput. Phys. Commun.* **2005**, *167*, 103.

[4] J. P. Perdew, K. Burke, M. Ernzerhof, *Phys. Rev. Lett.* **1996**, *77*, 3865.

[5] M. Guidon, J. Hutter, J. VandeVondele, *J. Chem. Theory Comput.* **2009**, *5*, 3010.

[6] G. Lippert, J. Hutter, M. Parrinello, *Mol. Phys.* **1997**, *92*, 477.

[7] J. VandeVondele, J. Hutter, *J. Chem. Phys.* **2007**, *127*, 114105.

[8] S. Goedecker, M. Teter, J. Hutter, *Phys. Rev. B* **1996**, *54*, 1703.

[9] D. M. Evans, T. S. Holstad, A. B. Mosberg, D. R. Småbråten, P. E. Vullum, A. L. Dadlani, K. Shapovalov, Z. Yan, E. Bourret, D. Gao, J. Akola, J. Torgersen, A. T. J. van Helvoort, S. M. Selbach, D. Meier, *Nat. Mater.* **2020**, *19*, 1195.

[10] M. Guidon, J. Hutter, J. Vandevondele, *J. Chem. Theory Comput.* **2010**, *6*, 2348.

[11] G. Henkelman, H. Jónsson, *J. Chem. Phys.* **2000**, *113*, 9978.

[12] G. Henkelman, B. P. Uberuaga, H. Jónsson, *J. Chem. Phys.* **2000**, *113*, 9901.

[13] P. E. Blöchl, *Phys. Rev. B* **1994**, *50*, 17953.

[14] G. Kresse, J. Furthmuller, *Phys. Rev. B* **1996**, *54*, 11169.

[15] G. Kresse, D. Joubert, *Phys. Rev. B - Condens. Matter Mater. Phys.* **1999**, *59*, 1758.

[16] S. Dudarev, G. Botton, S. Y. Savrasov, C. J. Humphreys, A. P. Sutton, *Phys. Rev. B - Condens. Matter Mater. Phys.* **1998**, *57*, 1505.

[17] S. H. Skjaervø, E. T. Wefring, S. K. Nesdal, N. H. Gaukås, G. H. Olsen, J. Glaum, T. Tybell, S. M. Selbach, *Nat.Commun.* **2016**, *7*, 13745.

[18] S. H. Skjærvø, D. R. Småbråten, N. A. Spaldin, T. Tybell, S. M. Selbach, *Phys. Revi. B* **2018**, *98*, 184102.

[19] K. A. Hunnestad, C. Hatzoglou, F. Vurpillot, I. E. Nylund, Z. Yan, E. Bourret, A. T. J. van Helvoort, D. Meier, *Mater. Charact.* **2023**, *203*, 113085.

[20] A. Minenkov, N. Šantić, T. Truglas, J. Aberl, L. Vukušić, M. Brehm, H. Groiss, *MRS Bull.* **2022**, *47*, 359.

[21] D. M. Evans, D. R. Småbråten, T. S. Holstad, P. E. Vullum, A. B. Mosberg, Z. Yan, E. Bourret, A. T. J. Van

Helvoort, S. M. Selbach, D. Meier, *Nano Lett.* **2021**, *21*, 3386.

[22] A. Ruff, Z. Li, A. Loidl, J. Schaab, M. Fiebig, A. Cano, Z. Yan, E. Bourret, J. Glaum, D. Meier, S. Krohns, *Appl. Phys. Lett.* **2018**, *112*, DOI 10.1063/1.5026732.

[23] L. Kuerten, S. Krohns, P. Schoenherr, K. Holeczek, E. Pomjakushina, T. Lottermoser, M. Trassin, D. Meier, M. Fiebig, *Phys. Revi. B* **2020**, *102*, 94108.

[24] Z. Chen, X. Wang, S. P. Ringer, X. Liao, *Phys. Rev. Lett.* **2016**, *117*, 1.

[25] S. Cheng, Q. Meng, M. G. Han, S. Deng, X. Li, Q. Zhang, G. Tan, G. A. Botton, Y. Zhu, *Adv. Electron. Mater.* **2019**, *5*, DOI 10.1002/aelm.201800827.
